# Effects of temperature and humidity on the efficacy of methicillin-resistant *Staphylococcus aureus* challenged antimicrobial materials containing silver and copper

**DOI:** 10.1111/j.1472-765X.2009.02637.x

**Published:** 2009-08

**Authors:** HT Michels, JO Noyce, CW Keevil

**Affiliations:** 1 Copper Development Association Inc.New York, NY, USA; 2 School of Biological Sciences, University of SouthamptonSouthampton, UK

**Keywords:** antimicrobial, efficacy, infection, MRSA, staphylococci, test methods

## Abstract

**Aims:**

To compare silver and copper, metals with known antimicrobial properties, by evaluating the effects of temperature and humidity on efficacy by challenging with methicillin resistant *Staphylococcus aureus* (MRSA)*.*

**Methods and Results:**

Using standard methodology described in a globally used Japanese Industrial Standard, JIS Z 2801, a silver ion-containing material exhibited >5 log reduction in MRSA viability after 24 h at >90% relative humidity (RH) at 20°C and 35°C but only a <0·3 log at ∼22% RH and 20°C and no reduction at ∼22% RH and 35°C. Copper alloys demonstrated >5 log reductions under all test conditions.

**Conclusions:**

While the high humidity (>90% RH) and high temperature (35°C) utilized in JIS Z 2801 produce measurable efficacy in a silver ion-containing material, it showed no significant response at lower temperature and humidity levels typical of indoor environments.

**Significance and Impact of the Study:**

The high efficacy levels displayed by the copper alloys, at temperature and humidity levels typical of indoor environments, compared to the low efficacy of the silver ion-containing material under the same conditions, favours the use of copper alloys as antimicrobial materials in indoor environments such as hospitals.

## Introduction

MRSA continues to be a major problem since its emergence in hospitals about three decades ago. In 2005, it was estimated that 94 650 patients in the United States had invasive MRSA infections. These infections resulted in 18 650 deaths (Klevens *et al.* 2007). The US Centers for Disease Control and Prevention indicates that this exceeded the number of fatalities due to AIDS. MRSA is a major health problem that is no longer confined to intensive care units (Klevens *et al.* 2007), but a serious hospital-acquired or nosocomial infection that has jumped to the general community. Although it is generally accepted that MRSA is spread by the hands of patients and medical staff (Cimolai 2008), there is growing evidence that environmental surfaces may also serve as an important reservoir for infectious bacteria (Casey *et al.* 2008), that may contribute to their dissemination.

MRSA infections continue to cause concerns, not only in the healthcare setting but also in farm animals. In both situations the emergence of MRSA is particularly problematic. In the Netherlands, a new strain of MRSA, related to pig and cattle farming, was reported, and a survey of pigs found that nearly 40% carried this clone (van Loo *et al.* 2003). In Korea, MRSA strains were found in chickens and dairy cows (Lee 2003). MRSA has become established in farm animals and has the potential to be spread from animals to farmers (Lee 2003), and then to the general public.

Community acquired MRSA is also a growing problem (Klevens *et al.* 2007). Those infected are generally younger than patients with hospital acquired MRSA. MRSA was found in individuals recently discharged from hospitals as well as others with no obvious risk factors. Transmission involves minor skin trauma, sharing sports and personal care equipment and being in crowded conditions and close quarters. Outbreaks have occurred in schools, professional and high school sports facilities, military training centers and prisons.

Antimicrobial active ingredients have been introduced into materials in order to reduce the ability of microbes to grow and/or survive on objects. The utilization of antimicrobial materials as environmental surfaces has the potential to reduce MRSA levels in clinical settings and beyond. A reduction in the level of MRSA on environmental surfaces may reduce the amount of bacteria subsequently available for transmission to humans when these surfaces are touched. Beyond copper alloys, which were registered by the US EPA (US Environmental Protection Agency 2008) as antimicrobial materials legally permitted to make public health claims, there are other materials, including those containing silver-ions that are purported to exhibit antimicrobial efficacy.

Silver ion-containing materials have demonstrable efficacy under the high temperature, 35°C, and high humidity conditions, >90% RH, prescribed by JIS Z 2801 (Japanese Standards Association 2000). This study was carried out to gain insight into the efficacy of the silver ion containing material at lower levels of temperature and humidity typical of indoor environments seen in hospitals. Copper alloys were used as a comparison because they display antimicrobial efficacy at lower levels of temperature and humidity typically found in hospitals (Noyce *et al.* 2006a).

## Materials and methods

Two commercial silver-containing materials were tested, but complete descriptions were not available because they are proprietary products. The first material, designated Ag-A for the purposes of this study, is described by its manufacturer as incorporating ‘silver ions in a zeolite carrier.’ Zeolites are aluminosilicate minerals with a microporous structure. The second material, designated Ag-B, consists of ‘silver ions as the active ingredient, in an organic matrix,’ according to its manufacturer. In both Ag-A and Ag-B, silver ion containing coatings were commercially applied to stainless steel substrates as a source for test samples.

The compositions of five copper alloys and the S30400 stainless steel experimental control are described by their UNS Number, a widely used alloy designation system. The compositions of copper alloys and stainless steel, as well as the silver content of one of the silver ion-containing materials, Ag-A, are presented in Table 1. The silver content of Ag-B was not provided, although it is believed to be nominally equivalent to Ag-A, which contains 2·5% Ag. It would be of scientific interest to also include a 99·9% fine silver in these tests. However, as a practical matter, fine silver is not used as structural materials because of its low strength and high cost.

**Table 1:** Nominal alloy composition (weight %)

UNS number: common name	Cu	Zn	Sn	Ni	Fe	Cr	P	Ag
C11000: copper	99·90							
C51000: phosphor bronze	95		5				0·2	
C70600: Cu-Ni	90			10				
C26000: cartridge brass	70	30						
C75200: Cu-Ni-Zn	65	17		18				
S30400: stainless steel				8	74	18		2·5
Ag-A*: proprietary product								2·5

*No UNS Alloy Number applies as Ag-A is not a metal alloy. Only the silver content of this proprietary silver ion-containing coating was provided.

All sample materials were cut into one of two sizes, either 1 × 1 cm or 2·54 × 2·54 cm squares. The alloy samples were degreased and cleaned by vortexing for 30 s in 10 ml of acetone containing 30 glass beads, 2 mm in diameter. After cleaning, the alloy samples were sterilized by ethanol immersion and Bunsen flaming and transferred to a covered plastic container prior to inoculation to prevent contamination. The silver ion-containing samples were cleaned and alcohol dipped but not degreased and flamed to avoid damaging the coatings.

In the first phase of testing, the objective was to evaluate efficacy as a function of exposure time to MRSA inoculums under typical indoor conditions. Tryptone soy broth was inoculated with MRSA (NCTC 10442), and incubated at 37°C for 16 h. This was followed by inoculation of triplicate coupons of each material for each data point, at a concentration of approximately 2 × 10^7^ CFU per coupon, as determined by serial dilution. These inoculated samples were exposed at 22 ± 2°C, or ∼22°C, and 50 ± 10% RH, or ∼50% RH for varying times ranging from 15 min to 6 h. After the exposure period, the organisms were removed from the samples by vortexing for 30 s in 10 ml of sterile phosphate buffered saline containing 20 glass beads, 2 mm in diameter. To ascertain the number of viable organisms removed from the samples, 100 μl was removed and serially diluted. Nutrient agar plates were inoculated with 50 μl of each dilution, spread evenly over the surface, incubated at 37°C for 18 h, and the number of CFU was counted and used to calculate the number of CFU/sample, as described in more detail elsewhere (Noyce *et al.* 2006a).

Antimicrobial materials, such as those containing silver ions, typically report results based on tests conducted for 24 h at a temperature of 35°C, and >90% RH as called for in the JIS Z 2801 standard. The required high humidity level is maintained by pressing a plastic film over the inoculums on the sample surface and placing it in a covered Petri dish to help retain moisture. In contrast, copper alloys are usually tested at typical laboratory environments of ∼20°C and ambient humidity levels that are much lower than >90% RH specified in JIS Z 2801.

In the second phase of testing, the objective was to evaluate efficacy after 24 h under JIS Z 2801 test conditions (Japanese Standards Association 2000), as well as at other levels of temperature and relative humidity. The actual JIS Z 2801 test was conducted for 24 h at 35 ± 2°C (∼35°C) and >90% RH. The tests were repeated at >90% RH, but at a lower temperature of 20 ± 2°C (∼20°C). In addition, two parallel series of tests were conducted at lower humidity levels found in the laboratory. These lower humidity tests were in air and no plastic film was used to maintain humidity. The first was at 35 ± 2°C at 15–24% RH and the second at 20 ± 2°C at 19–28% RH. For ease, 15–24% RH is shown as ∼20% RH, and the 19–28% RH is shown as ∼24% RH. Standard microbiological techniques, similar to those described above for the first phase of testing, were used to culture, recover and enumerate bacteria. In this phase of testing, Synthetic Broth AOAC was aseptically inoculated with MRSA (ATCC 33592), and incubated at 37°C overnight. The inoculums contained approximately 6 × 10^8^ CFU ml^−1^ of MRSA, as well as a soil load of 5% foetal bovine serum and 0·01% Triton X-100. Triplicate coupons of each material or alloy were inoculated and exposed for 24 h at the desired temperatures and relative humidity levels. After exposure, the remaining viable bacteria were then recovered, cultured and enumerated. The reductions in all other materials were measured relative to the number of survivors on the control material S30400 stainless steel at the end of the 24-h test period, under the test conditions of temperature and relative humidity.

## Results

**Figure 1:**
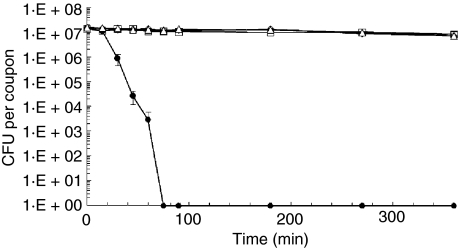
MRSA viability at ∼22°C and ∼50% RH on C11000 copper (•), two silver-ion containing materials, Ag-A (Δ), and Ag-B (◊) and S30400 stainless steel (□).

The objective of first phase of testing was to evaluate efficacy of silver ion-containing materials and copper, as a function of exposure time to MRSA inoculums. Specifically, the antimicrobial efficacy of the two previously defined silver ion-containing materials, Ag-A and Ag-B, as well as C11000 copper, was measured under typical laboratory temperatures of ∼22°C and ∼50% RH with S30400 stainless steel serving as the experimental control. The results of the initial study, presented in Fig. 1, show a seven log reduction in 75 min for C11000 copper, but no meaningful reduction was seen at 360 min in the two silver ion-containing materials, designated Ag-A and Ag-B as well as the experimental control, S30400 stainless steel. The two silver ion-containing materials were indistinguishable from each other, and showed no meaningful difference in efficacy from the experimental control, S30400 stainless steel, under the test conditions.

After completing the first phase of testing, a second phase of testing was conducted to better understand the efficacy claims of silver-ion containing materials and to gain additional insight into their antimicrobial efficacy over a range of temperature and humidity. This second phase involved testing one of the silver ion-containing materials, Ag-A, and five copper alloys, in accord with the JIS Z 2801 protocol, with S30400 stainless steel again serving as the experimental control. Additional, 24 h efficacy tests were conducted at the JIS Z 2801 humidity level of >90% RH, but at a lower temperature of ∼20°C. Two other 24 h efficacy tests were conducted at lower humidity levels, the first at ∼20% RH and ∼35°C and the second at ∼24% RH and ∼20°C. These results are presented in Table 2.

**Table 2:** Log_10_ reduction in live MRSA relative to the S304 stainless steel control after 24 h at >90% RH & ∼35°C, >90% RH & ∼20°C, ∼20% RH & 35°C, or ∼24% RH and ∼20°C

	>90% RH*	>90% RH	∼20% RH	∼24% RH
Materials	∼35°C	∼20°C	∼35°C	∼20°C
C11000: copper	>6·4	>6·1	>5·5	>5·9
C51000: phosphor bronze	>6·4	>6·1	>5·5	>5·9
C70600: Cu-Ni	>6·4	>6·1	>5·5	>5·9
C26000: cartridge brass	>6·3	>6·1	>5·5	>5·9
C75200: Cu-Ni-Zn	>6·4	>6·1	>5·5	>5·9
Ag-A†	>6·4	5·5	0	<0·2

* High humidity and temperature test conditions of JIS Z 2801 Protocol.

† Silver ion-containing coating.

When tested under the JISZ 2801 test conditions of ∼35°C and >90% RH, a >6·4 log drop was seen in the silver ion-containing material designated Ag-A, while a smaller 5·5 log drop was observed in the same material at ∼20°C and >90% RH. At the lower humidity levels, the silver ion-containing material, Ag-A, showed no reduction, or zero at ∼35°C, and ∼24% RH, and a very small <0·2 log reduction at ∼20°C and ∼24% RH. In contrast, at all combinations of humidity and temperature, all five of the copper alloys exhibited a >5·5 to >6·4 log reduction in viable MRSA after 24 h.

## Discussion

The data presented in Table 2 indicates the silver ion-containing material, Ag-A, exhibits efficacy under the high humidity and high temperature conditions, >90% RH and ∼35°C, of JIS Z 2801, as well as at >90% RH and ∼20°C. However, Ag-A exhibits no meaningful antimicrobial efficacy at ∼20% RH and 35°C, and more importantly at ∼24% RH and 20°C, where the latter is typical of indoor environments. This suggests that the JIS Z 2801 is not an appropriate methodology for evaluating the efficacy of antimicrobial materials intended for use in typical indoor environments. Results from JIS Z 2801 may potentially mislead consumers by giving them a false confidence because they may interpret that the efficacy is applicable to typical indoor environments, such as those found in hospitals.

The lack of efficacy of silver ion-containing materials was recently verified by Wood *et al.* (2007) in a hospital setting, which approximates the ∼24% RH and 20°C test conditions. It was determined that stethoscope protective diaphragm covers, made from silver ion-containing material Ag-A, had a mean colony count of 246·5 per sample, while the uncovered stethoscopes diaphragms had 71·4 colonies per sample (Wood *et al.* 2007). Thus the silver ion-containing protective covers, which were reported to be antimicrobial, were more heavily contaminated than the unprotected stethoscope diaphragms. This lack of efficacy in Ag-A may explain, in part, why antimicrobial silver ion-containing materials do not have US EPA registrations that permit public health claims. Materials like Ag-A can’t legally make public health claims in the US. However, they can make claims under a treated article exemption (US Environmental Protection Agency 2003). Treated article exemptions indicate that the active ingredient, silver, protects the silver ion-containing coating itself from deterioration or attack by bacteria.

In marked contrast to the silver ion-containing material, antimicrobial copper alloys, which have recently been registered as solid materials with public health claims (US Environmental Protection Agency. 2008), after demonstrating strong antimicrobial efficacy against five bacteria when tested according to three US EPA protocols (Michels and Anderson 2008). These five bacteria are *Staphylococcus aureus, Escherichia coli* O157 : H7, Methicillin-Resistant *S. aureus* (MRSA), *Enterobacter aerogenes* and *Pseudomonas aeruginosa.* Several studies that predate the EPA registration provided evidence that copper alloys have a strong antimicrobial efficacy on a range of bacteria as well as the one virus tested. These organisms include *Clostridium difficile* (Weaver *et al.* 2008), *E. coli* O157 (Wilks *et al.* 2005) and (Noyce *et al.* 2006b), *Listeria monocytogenes* (Wilks *et al.* 2006), Mycobacterium tuberculosis (Mehtar *et al.* 2008), and Influenza A (Noyce *et al.* 2007).

Although the mechanisms of bacterial inactivation on copper surfaces have not been elucidated, results generated to date suggest that bacteria are killed rapidly on dry copper surfaces. This fast action may, in part, explain the absence of significant copper resistant pathogenic bacteria. Copper has been widely used and in contact with humans for centuries which should have subjected bacteria to sublethal exposure. A preliminary study (Espirito Santo *et al.* 2008) indicated that bacteria do not have time to develop biofilms in dry conditions, and the stress and survival conditions are different from those of aqueous systems. One possible mechanism of toxicity is DNA damage. However, Macomber *et al.* (2007) reported, in a study on the toxicity of dry copper surfaces, that ‘it is clear that copper does not catalyze significant oxidative DNA damage *in vivo*; therefore, copper toxicity must occur by a different mechanism.’ Thus one may surmise that even if an organism has copper tolerance in aqueous environments, this may not allow them to survive on dry copper surfaces. Work on the mechanism of toxicity is continuing.

The strong and consistent efficacy seen in copper alloys against several types of bacteria indicates that this class of materials should be subjected to further evaluations in hospital settings. To fulfill that objective, coordinated clinical trials are underway in the intensive care units of three institutions in the US. The trial sites are at Memorial Sloan-Kettering Cancer Center in New York City, the Medical University of South Carolina and the Ralph H. Johnson VA Medical Center, both in Charleston, South Carolina, with funding provided through the US Department of Defense’s Telemedicine & Advanced Technology Research Center. Preliminary results from a clinical trial conducted at University Hospital NHS Foundation Trust in Birmingham, UK (Casey *et al.* 2008) indicated that all copper containing items have 90–100% fewer microorganisms than their control equivalents. This trial is also continuing.

It is anticipated that the results of these trials will further demonstrate that copper alloys have efficacy in hospital settings and can play a role in helping control the bacteria responsible for hospital acquired infections.
